# In Situ FTIR Spectroscopy for Scanning Accessible Active Sites in Defect-Engineered UiO-66

**DOI:** 10.3390/nano13101675

**Published:** 2023-05-18

**Authors:** Vera V. Butova, Videlina R. Zdravkova, Olga A. Burachevskaia, Andrei A. Tereshchenko, Pavletta S. Shestakova, Konstantin I. Hadjiivanov

**Affiliations:** 1 Institute of General and Inorganic Chemistry, Bulgarian Academy of Sciences, 1113 Sofia, Bulgaria; 2 The Smart Materials Research Institute, Southern Federal University, Rostov-on-Don 344090, Russia; 3 Institute of Organic Chemistry with Centre of Phytochemistry, Bulgarian Academy of Sciences, 1113 Sofia, Bulgaria

**Keywords:** adsorption, benzoic acid, carbon monoxide, CD_3_CN, missing linker defects, UiO-66

## Abstract

Three UiO-66 samples were prepared by solvothermal synthesis using the defect engineering approach with benzoic acid as a modulator. They were characterized by different techniques and their acidic properties were assessed by FTIR spectroscopy of adsorbed CO and CD_3_CN. All samples evacuated at room temperature contained bridging μ_3_-OH groups that interacted with both probe molecules. Evacuation at 250 °C leads to the dehydroxylation and disappearance of the μ_3_-OH groups. Modulator-free synthesis resulted in a material with open Zr sites. They were detected by low-temperature CO adsorption on a sample evacuated at 200 °C and by CD_3_CN even on a sample evacuated at RT. However, these sites were lacking in the two samples obtained with a modulator. IR and Raman spectra revealed that in these cases, the Zr^4+^ defect sites were saturated by benzoates, which prevented their interaction with probe molecules. Finally, the dehydroxylation of all samples produced another kind of bare Zr sites that did not interact with CO but formed complexes with acetonitrile, probably due to structural rearrangement. The results showed that FTIR spectroscopy is a powerful tool for investigating the presence and availability of acid sites in UiO-66, which is crucial for its application in adsorption and catalysis.

## 1. Introduction

**Figure 1:**
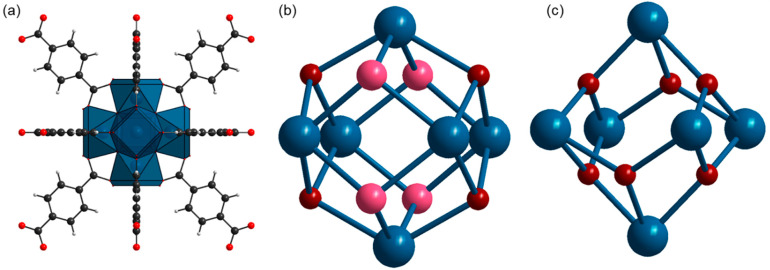
Model of the UiO-66 structure. Part (**a**) represents the coordination of the zirconium cluster Zr_6_O_4_(OH)_4_ with 12 BDC linkers. Blue polyhedra represent Zr coordination with O. (**b**) Zirconium cluster of unactivated UiO-66, Zr_6_O_4_(OH)_4_. (**c**) Zirconium cluster of activated UiO-66, Zr_6_O_6_. Gray spheres represent carbon; red spheres—oxygens from the linker carboxylic groups; dark red spheres—μ_3_-O; pink spheres—μ_3_-OH; blue spheres—Zr. The crystal structure model of UiO-66 is elaborated according to crystallographic data from ref. [24].

Metal–organic frameworks are porous materials that remain the focus of many researchers due to their tunable properties [1,2,3,4,5] and applications in various fields such as catalysis [6,7], gas storage [8,9,10], drug delivery [11,12,13], waste scavenging [14,15], etc. Among the wide variety of MOFs, UiO-66 is among the most promising due to its high stability and porosity and its unique (de)hydroxylation behavior [16,17]. Starting from the first report in 2008 [18], and in subsequent studies [19,20], UiO-66 has shown excellent potential in many applications. It is constructed of zirconium oxo clusters and 1,4-benzene dicarboxylate (BDC) linkers [21]. Their combination results in a cubic framework with two kinds of pores—octahedral and tetrahedral. Zirconium clusters consist of six zirconium ions connected via bridging oxygens and OH groups. Each cluster is coordinated to 12 BDC linkers (Figure 1a). Strong Zr–O bonds of highly covalent character and high connectivity are responsible for the extremely stable framework. The pores of the as-synthesized samples are filled with solvent molecules. Heating and degassing lead to the removal of guest molecules from the host structure, leaving empty accessible pores. After such treatment, the UiO-66 composition can be represented by the Zr_6_O_4_(OH)_4_(BDC)_6_ formula unit [22]. The zirconium ions in this case are connected via four μ_3_-O and four μ_3_-OH groups (Figure 1b). The latter provide acidic properties to the ideal UiO-66 framework. This feature applies to catalysis and separation. Further heating and outgassing lead to the dehydroxylation process. UiO-66 loses two water molecules, and each zirconium cluster retains only six μ_3_-O, corresponding to the Zr_6_O_6_(BDC)_6_ formula unit (Figure 1c). The dehydroxylation process results in a distortion of the zirconium cluster and a reduction in the Zr coordination number from 8 to 7 [22,23]. However, it has been reported [22] that the high packing of the zirconium cluster bonded to 12 linker units does not allow access to the metal centers.

In the typical synthesis of UiO-66, zirconium ions interact with water molecules, forming nuclei [25,26,27]. This step initiates a rapid increase in the cluster formation rate due to the autocatalytic nature of the process [28]. A large number of nuclei in the reaction mixture causes cascade aggregation. The next step is crystal growth. This process decreases the surface-to-volume ratio, stabilizing crystals by the attachment of monomer units [27,28]. In this case, nucleation dominates the precipitation process, resulting in polydisperse-aggregated UiO-66 nanoparticles. 

The defect engineering approach was proposed as a powerful tool for modifying the structure and properties of UiO-66 [29,30,31]. According to this approach, monocarboxylic acids act as modulators. These chemicals are added into the reaction mixture containing zirconium salt and linkers. Modulator molecules compete with the linkers for Zr coordination, shifting the equilibrium toward crystal growth. The UiO-66 precipitate, in this case, comprises monodisperse particles with improved morphology and porosity. A higher modulator concentration leads to the formation of larger crystals. On the other hand, this obstructs substitution from the linkers and, in the product part of the Zr sites, retains modulators in the coordination sphere instead of the linker. Since the modulators are monocarboxylic acids, which are monotopic ligands, they cannot bridge two zirconium clusters. This leads to the formation of defects. Two types of such defects were proposed for UiO-66. A missing-linker defect is formed when one linker molecule is displaced from the structure, resulting in defective sites in two opposite zirconium clusters. The other option is missing-cluster defects. This kind of defect is typical of the process when a high concentration of modulator obstructs the formation of a zirconium cluster, leading to a defect pore with vacancies on twelve neighboring zirconium clusters. Both the concentration of the modulator and its nature affect the formation of defects. It was reported that a higher acidity of the modulator molecule promotes its interaction with Zr^4+^ ions in the reaction mixture, enhancing the modulator effect [29]. After the synthesis, modulator molecules compensate zirconium sites in UiO-66. Some of them can be eliminated by a simple evacuation. For instance, Cao et al. [32] used acetic, pentanoic, n-caprylic, n-dodecanoic, n-myristic, n-pentacyclic and n-palmitic acids as modulators for UiO-66 synthesis. After activation at 250 °C, no peaks of the modulator were detected in the NMR spectra, indicating the complete removal of the modulator molecules. Vermoortele et al. [19] used trifluoroacetic acid for UiO-66 synthesis, and it was detached at 320 °C. However, in many cases, the modulator anions cannot be removed by thermovacuum treatment without structure collapse. Thus, an INS study indicated that acetate ions terminated defects in UiO-66 even after evacuation at 250 °C for 24 h [33]. Benzoate ions were also found in the UiO-66 structure when benzoic acid was used as a modulator [30,34]. 

Defective samples with benzoate ions coordinating zirconium in defect pores were used for further functionalization. For example, the benzoates in the defect pores of UiO-66 were replaced with amino-benzoates, providing a targeted modification of defect pores [35]. In contrast to the introduction of amino groups via linker molecules, such a procedure concentrates functional groups preferentially inside the crystals rather than on the surface. It was used to catch palladium precursors and obtain ultrasmall Pd nanoparticles inside the UiO-66 pores. In our previous work, we applied such a post-synthetic technique to modify MOFs of the UiO-66 family [36]. In the first step, defective samples with compensating benzoate ions were synthesized. The modulator molecules were then substituted with 3-phosphonopropionic acid to provide free accessible acidic COOH^−^ groups to defect pores. Shearer et al. [37] used a two-step post-synthetic functionalization for UiO-66. First, they replaced benzoate molecules with formate ones and then introduced the monoethanolamine moiety by grafting L-serine to the defect sites. 

The catalytic properties of UiO-66 are mainly connected with the existence of μ_3_-OH groups with Brønsted acidic properties and coordinatively unsaturated Zr^4+^ Lewis acid sites. However, it is not yet well established how the missing linker defects affect the acid sites of UiO-66. The aim of this work is to comparatively study, by means of IR spectroscopy, the evolution of acid sites of UiO-66 samples with and without missing linker defects. For this purpose, we investigated one defect-free sample and two samples with different defect concentrations. We have chosen benzoic acid as a modulator because, according to literature data, benzoates are thermally stable, and thus we eliminated the effect of the removal of the modulator linker during activation. By analyzing the evolution of the spectra during the evacuation at increasing temperatures, we made conclusions about the removal of admixtures and dehydroxylation of the samples, while the acidic properties were established by adsorption of two probe molecules with different basicity—CO and CD_3_CN. 

## 2. Materials and Methods

The starting materials zirconium tetrachloride (ZrCl_4_), 1,4-benzene dicarboxylic acid (H_2_BDC), dimethylformamide (DMF), benzoic acid (BA) and methanol were purchased from Alfa Aesar and used without additional purification. Distilled water was purified via a Simplicity UV ultrapure water system. For convenience, some characteristics of the samples studied, as well as their designations, which will be used further in the text, are summarized in Table 1. 

All samples were synthesized using a described procedure [36] (see Supplementary Materials, Part 1 for details). Typically, ZrCl_4_ was dissolved in DMF. Deionized water was added to this solution. For the UiO-66-10BA and UiO-66-60BA samples, BA was admixed and stirred at room temperature to obtain a clear solution. Finally, H_2_BDC was introduced and dissolved. The reaction mixture was placed in an oven for 24 h at 120 °C. After synthesis, the white precipitates were collected by centrifugation, washed twice with pure DMF and with methanol and dried at 60 °C overnight.

X-ray powder diffraction (XRD) profiles were registered on a D2 PHASER diffractometer (Bruker Corporation, Germany) in the 2θ range 5–90° with 0.01 step (CuK_α_, λ = 1.5417 Å). Profile analysis was performed using Jana2006 software (version 30/11/2014) [38]. 

Transmission electron microscopy (TEM) images were collected using a transmission electron microscope FEI Tecnai G2 Spirit TWIN (accelerating voltage—80 kV). 

ATR-IR spectra were measured ex situ on a Bruker Vertex 70 spectrometer in the range from 5000 to 500 cm^–1^ with a resolution of 1 cm^−1^, accumulating 64 scans. We used an MCT detector and a Bruker Platinum ATR attachment. 

Raman spectra were measured using an EnSpectr R532 Raman microscope (Enhanced Spectrometry Inc., Meridian, USA) equipped with a 532 nm laser. The ×20 objective was used, the exposure time was 1750 ms per spectrum and the resulting spectrum was averaged over eight measurements. The Raman shift was measured in the range of 4090–138 cm^−1^ with a resolution of 1.5 cm^−1^. The samples were placed on a blanc quartz slide as powder. Background correction implemented in EnSpectr software (app version 123123123, release date 23/06/2021) was performed to eliminate the luminescence signal. 

Nitrogen adsorption/desorption isotherms were recorded on an Accelerated Surface Area and Porosimetry analyzer ASAP 2020 (Micromeritics) at −196 °C. The specific surface area values were calculated according to the Brunauer–Emmett–Teller (BET) model [39]. The samples were degassed at 150 °C for 24 h under a dynamic vacuum before the measurement. 

The thermal gravimetric analyzer (Netzsch) was applied for thermogravimetric analysis (TGA) and differential scanning calorimetry (DSC). Samples in corundum crucibles were heated at a rate of 10 °C min^−1^ in the air flow. 

In situ FTIR spectra of the self-supporting pellets were recorded with a Nicolet 6700 FTIR spectrometer, accumulating 64 scans at a spectral resolution of 2 cm^−1^. Self-supporting pellets were prepared from the sample powders and treated directly in a purpose-made IR cell, allowing measurement at ambient and low (ca. −173 °C) temperatures. The cell was connected to a vacuum adsorption apparatus with a residual pressure below 10^−3^ Pa. Before the adsorption experiments, the samples were activated by evacuation at different temperatures. Adsorption was performed by introducing 5 mbar of CO (Merck, 99.5%) or CD_3_CN (Merck, deuteration degree 99.96%) directly into the IR cell, followed by dilution and evacuation for various times. 

## 3. Results and Discussion

### 3.1. Initial Characterization of the Samples

#### 3.1.1. Structural Characterization and Porosity

**Figure 2:**
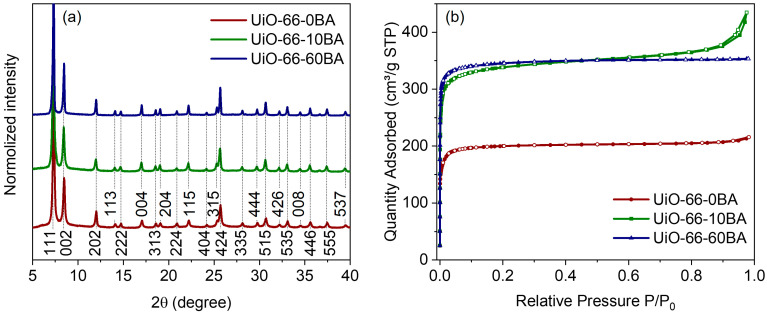
(**a**) XRD patterns of the UiO-66 samples. Profiles were shifted along the intensity axis for better representation. Numbers designate Miller indices. (**b**) N_2_ sorption isotherms. Adsorption branches of isotherms are represented by lines with filled markers; desorption ones are shown by lines with empty markers.

All synthesized samples were characterized as cubic UiO-66 phase with an Fm-3m space group (Figure 2a). The profile analysis revealed an expansion of the unit cell from UiO-66-0BA to UiO-66-10BA and the UiO-66-60BA sample (see details in Supplementary Materials, Table S1, Figure S1). This is attributed to an increased concentration of the missing linker defect. Compensation ion repulsion led to an increase in lattice parameters [40]. When the concentration of defects is rather high, they can join and form a missing-cluster defect. As reported [29,40], if UiO-66 misses a quarter of its clusters, the defects formed correlate to nanoregions of the reo topology. In this case, the defects do not lead to unit cell expansion and the PXRD profiles contain forbidden (100) and (110) reflections in the low-angle region [41,42]. We did not observe such features, which indicated the statistical distribution of defects.

Low-temperature N_2_ adsorption–desorption isotherms are provided in Figure 2b. All samples demonstrated Type I isotherms typical of microporous materials [43]. The isotherm of the UiO-66-10BA sample additionally contained a hysteresis loop. This feature is associated with capillary condensation of N_2_ in mesocavities between uniform nanoparticles in agglomerates. The specific surface areas were estimated as 795, 1331 and 1392 m^2^ g^−1^ for the UiO-66-0BA, UiO-66-10BA and UiO-66-60BA samples, respectively (see details in Supplementary Materials, Table S2, Figure S2). The UiO-66-0BA sample had a lower porosity than the theoretically predicted value of 1241 m^2^ g^−1^ [30]. This may indicate an admixture of non-porous phase or blocking-pore imperfections in the framework. In contrast, the UiO-66-10BA and UiO-66-60BA samples exhibited higher porosity than the ideal UiO-66, indicating defects in the structure. 

**Figure 3:**
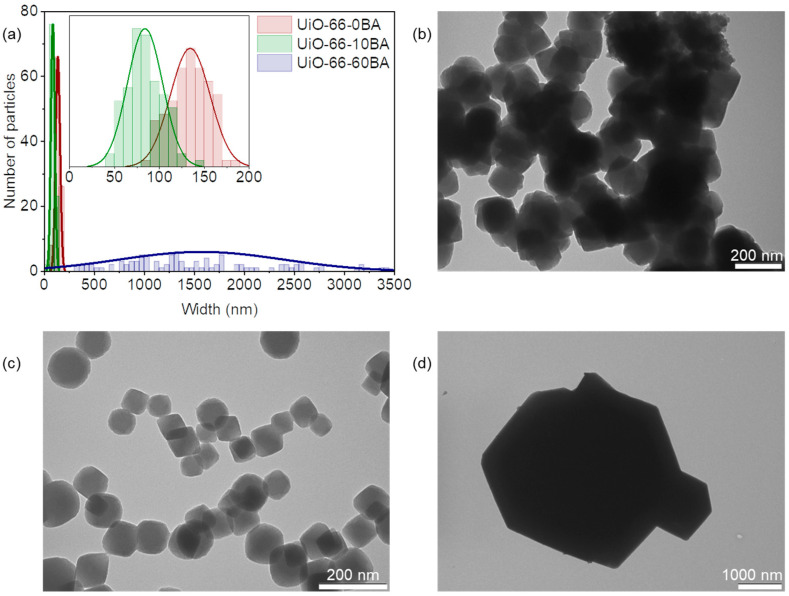
(**a**) Particle size distribution of UiO-66 samples according to TEM data (the inset shows the magnified part corresponding to data for UiO-66-0BA and UiO-66-10BA samples). Representative TEM images of UiO-66-0BA (**b**), UiO-66-10BA (**c**), and UiO-66-60BA (**d**) samples.

The modulator additive affected the particle size and morphology of the UiO-66 samples (Figure 3). The UiO-66-0BA sample comprised aggregated nanoparticles of about 130 nm. Ten equivalents of BA directed the synthesis toward forming monodispersed nanoparticles of about 80 nm without significant aggregation (UiO-66-10BA sample). UiO-66-60BA consists of polydispersed well-shaped crystals ranging from 300 up to 3000 nm. The same trend was previously observed for MOFs of the UiO-66 family [8,31]. The precipitate formation can be considered a combination of nucleation and crystal growth [44]. According to the observed trend, BA shifted the equilibrium toward crystal growth, resulting in larger crystals. Conversely, in the case of the UiO-66-0BA sample, nucleation-dominated phase formation leads to small crystals with lower porosity. 

#### 3.1.2. Thermal Analysis

**Figure 4:**
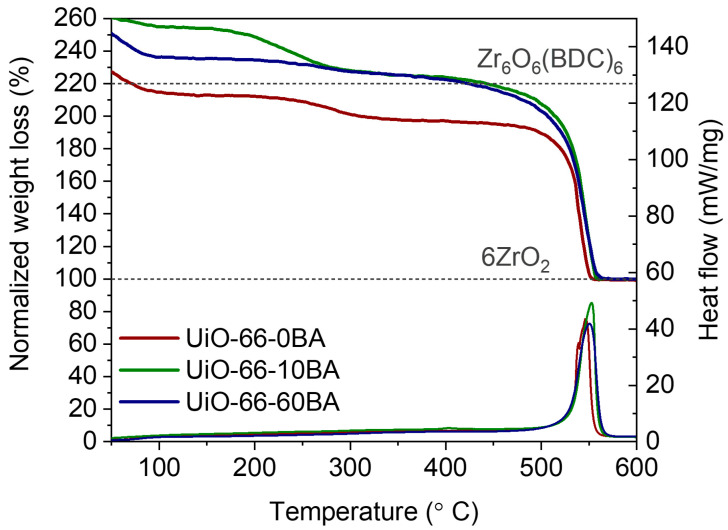
Normalized TGA and DSC curves of UiO-66 samples. Gray dashed lines correspond to 6 mols of ZrO_2_ and 1 mol of UiO-66 according to Zr_6_O_6_(C_8_H_4_O_4_)_6_ formula unit.

Figure 4 represents the TGA/DSC curves referring to UiO-66’s decomposition in air flow. TGA curves were normalized according to the reported procedure [22] (see details in Supplementary Materials, Figure S3, Table S3). The TGA curves of all samples contained three main steps. The first weight loss in the temperature range from 50 to 100 °C corresponds to the evacuation of physisorbed water molecules. The second weight loss occurs at 250–350 °C and is due to the elimination of DMF and dehydroxylation associated with the removal of two H_2_O molecules bonded to the zirconium cluster. The most pronounced step in the TGA curve at temperature 500–600 °C relates to the framework decomposition according to the following equation:

Zr_6_O_6_(C_8_H_4_O_4_)_6_ + 45O_2_ = 6ZrO_2_ + 48CO_2_ + 12H_2_O.

This process is exothermic, with a positive peak in the DSC curve. The theoretical weight loss of ideal UiO-66 with formula unit Zr_6_O_6_(C_8_H_4_O_4_)_6_ is 54.6%. UiO-66-0BA exhibited lower weight loss (49.2%). The uncompensated defects in UiO-66’s structure can reduce weight loss due to lower organic content. On the other hand, the presence of ZrO_2_ nuclei increases solid residual weight, reducing weight loss as well. The UiO-66-10BA and UiO-66-60BA samples demonstrated a higher weight loss than that of ideal defect-free UiO-66. The values were estimated to be 55.5 and 55.9% for the UiO-66-10BA and UiO-66-60BA samples, respectively. From one point of view, missing linker defects reduce weight loss. However, benzoate anions coordinated to Zr ions can overcompensate this effect. 

#### 3.1.3. Vibrational Spectroscopy

**Figure 5:**
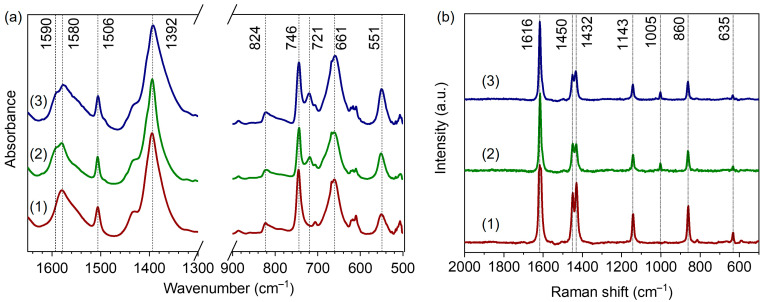
Selected regions of IR (**a**) and Raman (**b**) spectra of UiO-66-0BA (1), UiO-66-10BA (2), and UiO-66-60BA (3) samples.

Figure 5a represents the DRIFTS spectra of our UiO-66 samples in the fingerprint region. The two intense bands at 1580 and 1392 cm^−1^ are attributed to asymmetric and symmetric stretching vibrations of terephthalate linker carboxylate groups, respectively [18,45,46]. The C=C vibrations of the benzene rings gave rise to a sharp band at 1506 cm^−1^ [45]. The peaks at 746 and 824 cm^−1^ are associated with vibrations of the zirconium cluster in the UiO-66 framework, Zr–μ_3_-OH stretching and OH bending modes, respectively [46]. The FTIR spectra of UiO-66-10BA and UiO-66-60BA samples synthesized with the addition of benzoic acid contained peaks associated with benzoates: 1590, 1174, 1072, 934 and 721 cm^−1^ [30,47,48,49] (see also Supplementary Materials, Figure S4). These results indicate that benzoate ions compensate Zr sites in defect pores. Namely, the shoulder at 1590 cm^−1^ was assigned to the aromatic C–C stretching modes of benzoate. The β(C–H) vibrations of the bonded modulator gave rise to peaks at 1072 and 934 cm^−1^ [48]. Peaks at 762 and 721 cm^−1^ corresponded to out-of-plane symmetric deformations of the COO^−^ group and out-of-plane C–H vibrations of bonded benzoate [47,48].

The Raman spectra of the samples are presented in Figure 5b. The band at 1616 cm^−1^ is attributed to the C=C stretching modes of the benzene ring [30]. The doublet at 1450 and 1432 cm^−1^ corresponds to the in-plane stretching of BDC carboxylic groups. Breathing and deformation vibrations of terephthalate phenyl rings gave rise to bands at 1143 and 635 cm^−1^, respectively [30]. The band at 860 cm^−1^ was assigned to C–N stretching modes of residual DMF [50]. In good agreement with the IR results, the Raman spectra of the UiO-66-10BA and UiO-66-60BA samples contained a peak at 1005 cm^−1^ associated with the deformation modes of the benzene ring in benzoate ions [30]. 

To quantify the amount of benzoate anions in the different samples, we used NMR. Details are provided in the Supplementary Materials (Figure S5 and the corresponding text). According to the data obtained, the BDC:BA ratio was estimated to be 1:0, 1:0.5 and 1:0.6 for the samples UiO-66-0BA, UiO-66-10BA and UiO-66-60BA, respectively.

In summary, initial characterization revealed that all samples were of the UiO-66 cubic phase. The introduction of defects resulted in a small increase in the lattice constant, attributed to the repulsion between the compensating ions. The introduction of the modulator affected the crystal morphology of the UiO-66 samples. Thus, UiO-66-0BA and UiO-66-10BA comprised nanoparticles. The former sample exhibited significant aggregation, while UiO-66-10BA was composed of monodisperse well-shaped crystals of about 80 nm. The UiO-66-60BA sample contained well-shaped octahedral microcrystals. The modulator additive also increased the porosity from 795 m^2^ g^−1^ (corresponding to UiO-66-0BA) to 1331 and 1392 m^2^/g for the UiO-66-10BA and UiO-66-60BA samples, respectively. IR and Raman spectra revealed DMF molecules in all as-synthesized samples. The UiO-66-10BA and UiO-66-60BA samples, along with DMF, contained benzoate ions. We suppose that the latter compensated for zirconium sites in defect pores.

### 3.2. In Situ FTIR Spectroscopy

The in situ FTIR experiments were performed with self-supporting pellets, which led to a very high intensity of the principal bands. As a result, the exact positions of some of them cannot be precisely determined due to instrumental limitations. On the other hand, the bands of adsorbed species appeared with optimal intensities. 

#### 3.2.1. Activation of the Samples

**Figure 6:**
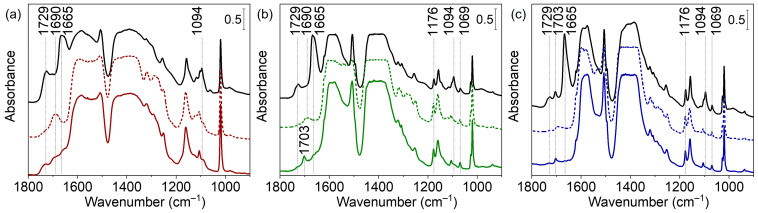
FTIR spectra of UiO-66-0BA (black and red lines) (**a**), UiO-66-10BA (black and green lines) (**b**), and UiO-66-60BA (black and blue lines) (**c**) samples evacuated at room temperature (black lines), at 250 °C (dashed lines), rehydrated and then evacuated at room temperature (solid lines). Spectra are provided in the 900–1800 cm^−1^ region.

To establish the temperature at which sorbed water and residual species are removed, we evacuated the samples at increasing temperatures. Consider first the bands characterizing residual DMF. Free DMF molecules give rise to several bands, among them at 1679 cm^−1^ (C=O stretching) and 1095 cm^−1^ (H_3_C–N stretching). With our RT evacuated samples, we detected these bands at 1665 and 1094 cm^−1^ (Figure 6, Supplementary Materials, Figure S6) [51]. The large shift of ν(CO) suggests that DMF is bound through the C=O groups. Evacuation at increasing temperatures resulted in a decrease in intensity and the ultimate disappearance of the DMF bands. The most pronounced band at 1665 cm^−1^ disappeared at 225 °C (UiO-66-0BA sample), 200 °C (UiO-66-10BA sample) and 175 °C (UiO-66-60BA sample) (see Supplementary Materials, Figure S6). Thus, it appears that the removal of DMF molecules is promoted by the presence of benzoates. The presence/removal of other minor admixtures is discussed in the SI as it is not directly relevant to the purpose of this study (Supplementary Materials, Figure S8a–c). 

**Figure 7:**
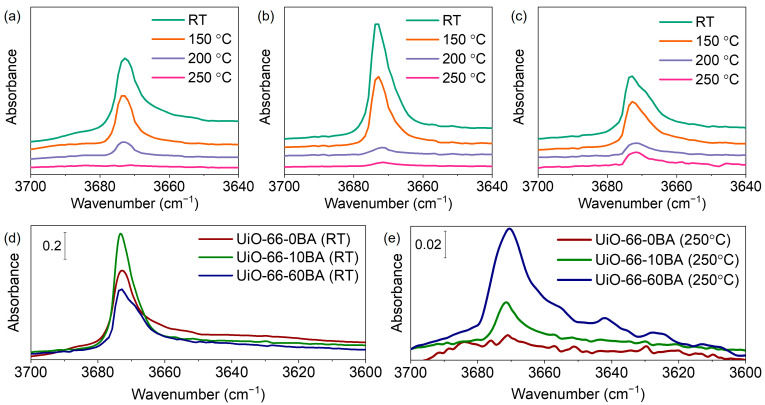
FTIR spectra of UiO-66-0BA (**a**), UiO-66-10BA (**b**), and UiO-66-60BA (**c**) samples during evacuation from room temperature (bottom spectra) up to 250 °C (top spectra). Spectra are provided in the 3700–3640 cm^−1^ region. FTIR spectra of UiO-66 samples evacuated at RT (**d**) and at 250 °C (**e**).

After evacuation of the as-prepared samples at RT, all spectra contained bands at 3672–3678 cm^−1^ associated with vibrations of isolated μ_3_-OH groups (Supplementary Materials, Figures S7 and S8d–f). The position was slightly shifted compared to the case of pure material, which is attributed to interaction with residual DMF. So, we followed the spectra of the DMF-free samples (evacuated at 275 °C and then rehydroxylated). In this case, the hydroxyl bands are not affected by guest molecules in the pores (Figure 7). It was reported that defect pores are larger than regular ones and restrain small molecules more weakly, which results in the easier evacuation of physisorbed water [30]. The intensity of the μ_3_-OH peaks decreased with the increase in BA concentration (Figure 7d). The dehydroxylation process is associated with splitting two water molecules from the zirconium cluster—transforming two μ_3_-OH-groups to μ_3_-O and removing two μ_3_-OH-groups as parts of water molecules. As theoretically predicted, this process becomes easier with neighboring linker deficiencies [52]. We hypothesized that missing-linker defects would lead to a lower concentration of μ_3_-OH-groups in UiO-66-10BA and UiO-66-60BA samples due to their partial evacuation at room temperature. Subsequent thermovacuum treatment resulted in the reduced intensity of these bands, indicating dehydroxylation of the Zr_6_O_4_(OH)_4_ cluster and the formation of Zr_6_O_6_ with bridging μ_3_-O groups (Figure 7a–c).

#### 3.2.2. Adsorption of CO at −173 °C

CO is one of the most used IR probe molecules for the determination of surface acidity [53,54,55]. It can be coordinated to metal cations, and, provided no back π-bond is formed, the CO stretching frequency is blue-shifted. A similar interaction occurs with the protons of the OH groups. In this case, an additional effect is registered, i.e., the OH stretching modes are red-shifted, and the higher the hydroxyl acidity, the larger the shift.

**Figure 8:**
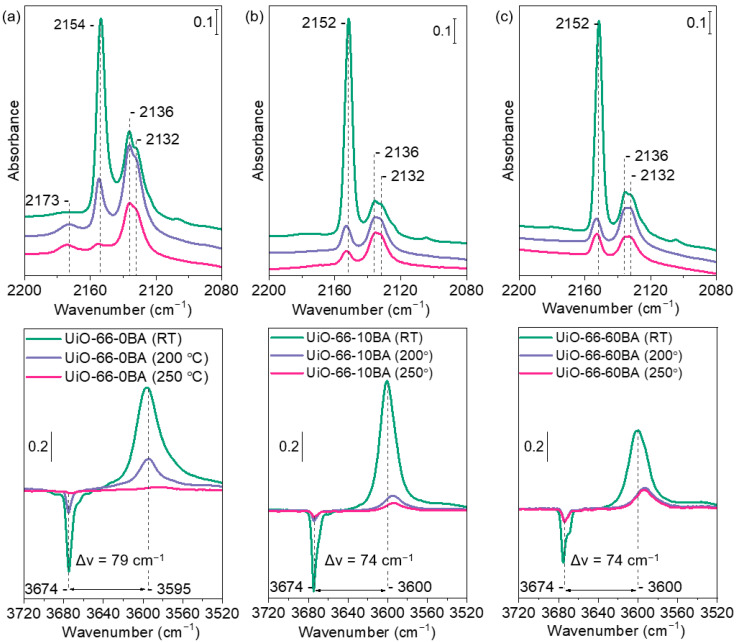
FTIR spectra registered after low-temperature adsorption of CO (1 mbar equilibrium pressure) on UiO-66-0BA (**a**), UiO-66-10BA (**b**), and UiO-66-60BA (**c**) samples evacuated at room temperature/200 °C/250 °C. The bottom parts show difference spectra.

After activation at 275 °C, the samples were rehydrated by contacting them with 5 mbar water vapor, followed by evacuation at different temperatures. The surface acidity of the UiO-66 samples after each evacuation treatment was assessed by using CO as a probe molecule. Figure 8 represents the changes occurring with the FTIR spectra of UiO-66 samples upon exposure to 1 mbar of CO at −173 °C. The evolution of the spectra during the decrease in CO coverage is presented in the Supplementary Materials, Figures S9–S11. 

The exposure of all samples to CO leads to the appearance of several bands in the carbonyl stretching region. Bands at 2154 cm^−1^ (UiO-66-0BA sample) and 2152 cm^−1^ (UiO-66-10BA and UiO-66-60BA samples) are associated with CO molecules that are C-bonded to μ_3_-OH groups. The slightly higher position of the band registered with UiO-66-0BA indicates a slightly higher acidity of the hydroxyls. Indeed, the υ(OH) modes with this sample are shifted upon CO adsorption by −79 cm^−1^, while the shift with the other two samples is −74 cm^−1^, confirming the slightly lower acidity of the respective hydroxyls.

Two bands at 2136 and 2132 cm^−1^ corresponded to physically adsorbed CO. A weak feature at ca 2110 cm^−1^ is attributed to O-bonded CO (see Supplementary Materials, Figure S9).

Evacuation at elevated temperatures leads to a substantial loss of OH groups and indicates a gradual transformation of Zr_6_O_4_(OH)_4_ clusters into Zr_6_O_6_. Accordingly, the bands at 2154–2152 cm^−1^, registered after subsequent CO adsorption, appeared with reduced intensities. Note that the acidity of the hydroxyls in the samples with a modulator slightly increases as their concentration decreases. An important observation is that a band at 2173 cm^−1^ was also observed, but only with the UiO-66-0BA sample evacuated at 200 and 250 °C. This band is attributed to Zr^4+^-CO species and indicates the existence of some accessible defect Zr sites in this sample. These sites are not seen with the sample evacuated only at RT because they are blocked by water molecules. 

#### 3.2.3. Adsorption of CD_3_CN

Another probe molecule widely used to evaluate surface acidity is CD_3_CN [51,56,57]. It usually forms H-bonds with hydroxyl groups which, as in the case of CO, leads to a red shift of the OH stretching modes. Because of the higher basicity of CD_3_CN compared to CO, the shift is larger. The binding of acetonitrile to hydroxyl groups leads to a blue shift of the ν(CN) modes. CD_3_CN can also be coordinated to unsaturated metal cations, and this is reflected in a larger blue shift of the ν(CN) vibrations.

**Figure 9:**
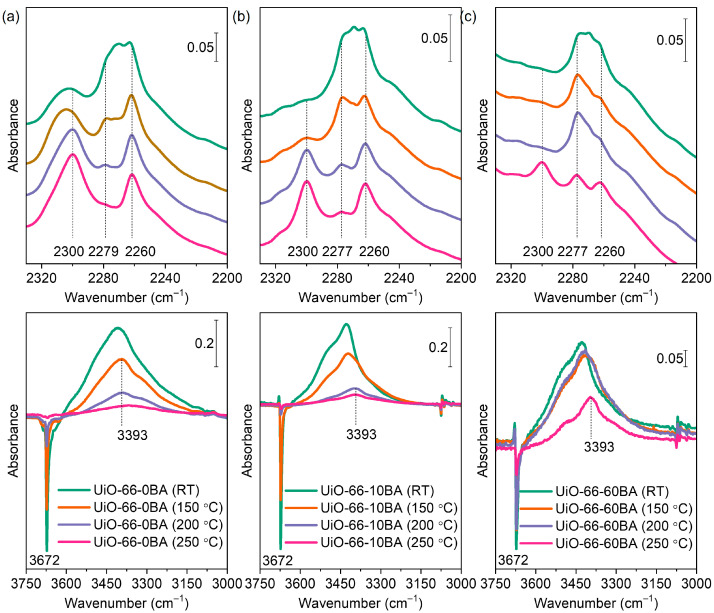
FTIR spectra of CD_3_CN (2 mbar equilibrium pressure) adsorbed on UiO-66-0BA (**a**), UiO-66-10BA (**b**), and UiO-66-60BA (**c**) samples evacuated at room temperature/150 °C/200 °C/250 °C. The bottom parts show difference spectra.

Figure 9 presents the FTIR spectra of UiO-66 samples evacuated at various temperatures and exposed to CD_3_CN vapor (see also Supplementary Materials, Figure S12). 

Evacuation at room temperature does not lead to dehydroxylation of the zirconium clusters in UiO-66. After such treatment, it contained four bridging oxygens and four hydroxyl bridging groups according to the formula unit Zr_6_O_4_(OH)_4_(BDC)_6_. A band at 2260 cm^−1^ was registered after CD_3_CN adsorption on all samples evacuated at ambient temperature and is attributed to non-specifically adsorbed (liquid-like) CD_3_CN. Another band at 2279 cm^−1^ (UiO-66-0BA sample) or 2277 cm^−1^ (UiO-66-10BA and UiO-66-60BA samples) is associated with CD_3_CN interacting with hydroxyl groups. For comparison, similar complexes formed with the more acidic silanol groups in silica and zeolites are detected at ca. 2290 cm^−1^ [56,58]. In the hydroxyl stretching region, the OH bands are red-shifted by 250–280 cm^−1^, which is consistent with the acidity measured by CO. Finally, a band at 2305 cm^−1^ was detected, but only with the UiO-66-0BA sample. This band is typical of CD_3_CN coordinated to Lewis acid sites and its appearance indicates the existence of accessible Zr^4+^ cations. Note that such sites were evidenced in this sample by CO adsorption, but only after evacuation at T ≥ 200 °C. However, CD_3_CN is a strong enough base to displace water coordinated to Zr^4+^ sites. Moreover, it was in great excess in our experiments. This explains why these sites were monitored by CD_3_CN. 

As already noted, evacuation at elevated temperatures leads to gradual dehydroxylation of the zirconium clusters. When the sample is fully dehydroxylated, the zirconium cluster has lost two H_2_O molecules and is transformed into Zr_6_O_6_(BDC)_6_ with six bridging oxygen atoms. This transformation was traced by the gradual disappearance of the μ_3_-OH band at 3676 cm^−1^ in the FTIR spectra (Figure 7). Consequently, the bands of CD_3_CN adsorbed on OH groups also vanished. Here, again, a slight increase in the acidity of hydroxyls with a decrease in their population was detected. We also note that dehydroxylation of the UiO-66-BA sample is limited even after evacuation at 250 °C, which is most likely related to the large particles of this sample and diffuse limitations. 

It should be emphasized that the modulator-containing samples evacuated at RT do not contain open Zr^4+^ sites. However, parallel with the dehydroxylation process, a band at 2300 cm^−1^, due to a second type of Zr^4+^–NCCD_3_ species, was detected. The C–N stretching frequency indicates these sites are characterized by a lower Lewis acidity as compared to the sites observed with the UiO-66-0 sample evacuated at RT (CD_3_CN band at 2305 cm^−1^). Similar conclusions are consistent with the spectra of the UiO-66-0 sample as well. In this case, the 2300 cm^−1^ band of adsorbed acetonitrile appears on samples evacuated at T ≥ 200 °C and is superimposed onto the band at 2305 cm^−1^. The results indicate that, in agreement with earlier results [51], the dehydroxylation of UiO-66 leads to the creation of Zr^4+^ sites that are able to adsorb relatively strong bases. However, these Zr^4+^ sites were not observed when the weak base CO was used as a probe molecule (Figure 8). These results strongly indicate that the Zr^4+^ sites in the dehydroxylated cluster are coordinatively saturated or inaccessible. However, some structural rearrangement occurs to allow the adsorption of strong bases on the zirconium sites. Most likely, the coordinative vacancies are located at the same cations where the OH groups have been bonded. 

Finally, we underline that all processes are fully reversible. The evacuation of acetonitrile results in its disappearance, while successive hydration restores the μ_3_-OH groups.

To summarize, samples were evacuated at 275 °C to remove residual DMF and then rehydrated. Subsequent evacuation at RT removes all adsorbed water while the µ_3_–OH groups (ca. 3674 cm^−1^) remain intact. When CO was adsorbed at −173 °C, a band at 2154–2152 cm^−1^ due to OH–CO adducts was registered with all samples. Simultaneously, a red shift of the OH modes was observed (Δν = −74 to −79 cm^−1^). Similar results were obtained using CD_3_CN as a probe: the appearance of a ν(C–N) band at ca. 2300 cm^−1^ (OH–NCCD_3_ complexes) and a red shift of the OH modes by ca. 280 cm^−1^. However, a band at 2300 cm^−1^, indicative of Zr^4+^–NCCD_3_ species, was also detected but only with the UiO-66(0) sample. This implies the existence of open Zr^4+^ sites in this sample.

Evacuation at higher temperatures leads to a progressive decrease in the intensity of the OH bands, and their intensity is very low with the 250 °C evacuated samples. Consequently, when CO was adsorbed on samples evacuated at this temperature, the intensity of the OH–CO band at 2154 cm^−1^ was also low. Noticeably, a band due to Zr^4+^–CO species (2173 cm^−1^) was detected, but again only with the UiO-66(0) sample. When CD_3_CN was used as a probe, the spectral signatures of the complexes associated with OH bands again had low intensity. However, the bands at ca. 2300 cm^−1^ and related to open Zr^4+^ sites were registered with all samples. 

## 4. Conclusions

UiO-66, which has no missing linker defects, possesses ordinary-defect Zr^4+^ sites. They remain blocked by H_2_O after RT evacuation and, therefore, cannot be detected by CO. However, being a stronger base, CD_3_CN can replace water and thus observe these Zr^4+^ sites. Samples with missing linker defects appear to be characterized by a lack of ordinary defect Zr^4+^ sites, likely due to the saturation of the latter by the modulator.

Another observation is that the removal of the OH groups also creates Zr^4+^ sites that can be monitored by CD_3_CN but not by CO. It is expected that the ideal dehydroxylated zirconium node should not contain open Zr^4+^ sites. However, due to the high basicity of CD_3_CN, we propose that it can form complexes with dehydroxylated sites, probably via some structural changes. The results show that combining different probe molecules allows crucial complementary information to be obtained. 

## Figures and Tables

**Table 1:** Some details on the synthesis of UiO-66-0BA, UiO-66-10BA and UiO-66-60BA samples.

Sample	Molar Ratio					Synthesis Conditions	
	ZrCl_4_	H_2_BDC	H_2_O	BA	DMF	Temperature, °C	Time, h
UiO-66-0BA	1	1	3	0	300	120	24
UiO-66-10BA	1	1	3	10	300	120	24
UiO-66-60BA	1	1	3	60	300	120	24

## Data Availability

The data presented in this study are available in the article and the supplementary material.

## Supplementary Materials

The following supporting information can be downloaded at https://www.mdpi.com/article/10.3390/nano13101675/s1, Synthesis details; Figure S1: XRD patterns; Table S1: Results of XRD profile analysis; Figure S2: BET surface area plots; Table S2: Details of porosity calculations. Figure S3: TGA and DTG curves; Table S3: TGA data; Figure S4: ^1^H NMR spectra; Figures S5 and S6: FTIR spectra of samples evacuated at different temperatures; Figure S7: FTIR spectra of rehydrated and evacuated samples; Figure S8: FTIR spectra of CO adsorbed at −173 °C on samples evacuated at RT; Figure S9: FTIR spectra of CO adsorbed at −173 °C on samples evacuated at 200 °C; Figure S10: FTIR spectra of CO adsorbed at −173 °C on samples evacuated at 250 °C; Figure S11: FTIR spectra of CD_3_CN adsorbed on the samples [59,60,61,62].
