# Supplementary material for: In Situ FTIR Spectroscopy for Scanning Accessible Active Sites in Defect-Engineered UiO-66
Source: Nanomaterials (Basel). 2023 May 18;13(10):1675. doi: 10.3390/nano13101675 (PMC10222444; doi:10.3390/nano13101675)
Supplement: Supplementary file 1 [file nanomaterials-13-01675-s001.zip › nanomaterials-2396613-supplementary.pdf]

nanomaterials

Supporting information

In-situ FTIR spectroscopy for scanning accessible active sites in defect engineered UiO-66

Vera V. Butova[1,2*], Videlina R. Zdravkova [1], Olga A. Burachevskaia[2], Andrei A. Tereshchenko[2], Pavletta S. Shestakova[3], Konstantin I. Hadjiivanov[1*]

[1] Institute of General and Inorganic Chemistry, Bulgarian Academy of Sciences, Sofia 1113, Bulgaria
[2] The Smart Materials Research Institute, Southern Federal University, Rostov-on-Don 344090, Russia
[3] Institute of Organic Chemistry with Centre of Phytochemistry (IOCCP) Bulgarian Academy of Sciences, Sofia 1113, Bulgaria

* Correspondence: VVB: v.butova@svr.igic.bas.bg; KIH: kih@svr.igic.bas.bg

Content

- Synthesis 1
- XRD 2
- N_2 adsorption 3
- TGA 4
- IR 5
- NMR 6
- FTIR, activation 6
- FTIR CO 8
- FTIR, CD_3CN 10

Synthesis

- $ZrCl_4$ (0.2512 g) was dissolved in 25 ml of DMF.
- Deionized water (58.3 µl) was added under magnetic stirring.
- 1.3151 g of BA (for UiO-66-10BA sample) / 7.8904 g of BA (for UiO-66-60BA sample) was added to the reaction mixture and stirred until clear solution.
- H_2BDC (0.1789 g) was added under magnetic stirring.
- The reaction mixture was placed into the preheated oven at 120 °C for 24 h.
- The vessel cooled down naturally.
- The precipitate was separated using a centrifuge at 9000 rpm for 15 minutes.
- The white precipitate was placed into the 25 ml of clean DMF and mixed using a rotary shaker for 12 h.
- The precipitate was separated using a centrifuge at 9000 rpm for 15 minutes.
- The white precipitate was placed into the 25 ml of clean DMF and mixed using a rotary shaker for 12 h.
- The precipitate was separated using a centrifuge at 9000 rpm for 15 minutes.
- The white precipitate was placed into 25 ml of clean methanol and refluxed for 7 h.
- The precipitate was separated using a centrifuge at 9000 rpm for 15 minutes.
- The precipitate was dried at 60 °C overnight.

XRD

Table S1. Results of XRD profile analysis.

Sample	Space group	a, Å	V, Å³	Agreement factors			
				GOF	R	wR	wR_{exp}
UiO-66 0BA	Fm-3m (225)	20.7459(4)	8928.9(3)	1.46	0.0604	0.0766	0.0524
UiO-66 10BA		20.7683(3)	8957.8(2)	1.47	0.0634	0.0834	0.0568
UiO-66 60BA		20.7518(3)	8936.5(3)	2.59	0.1015	0.1350	0.0521

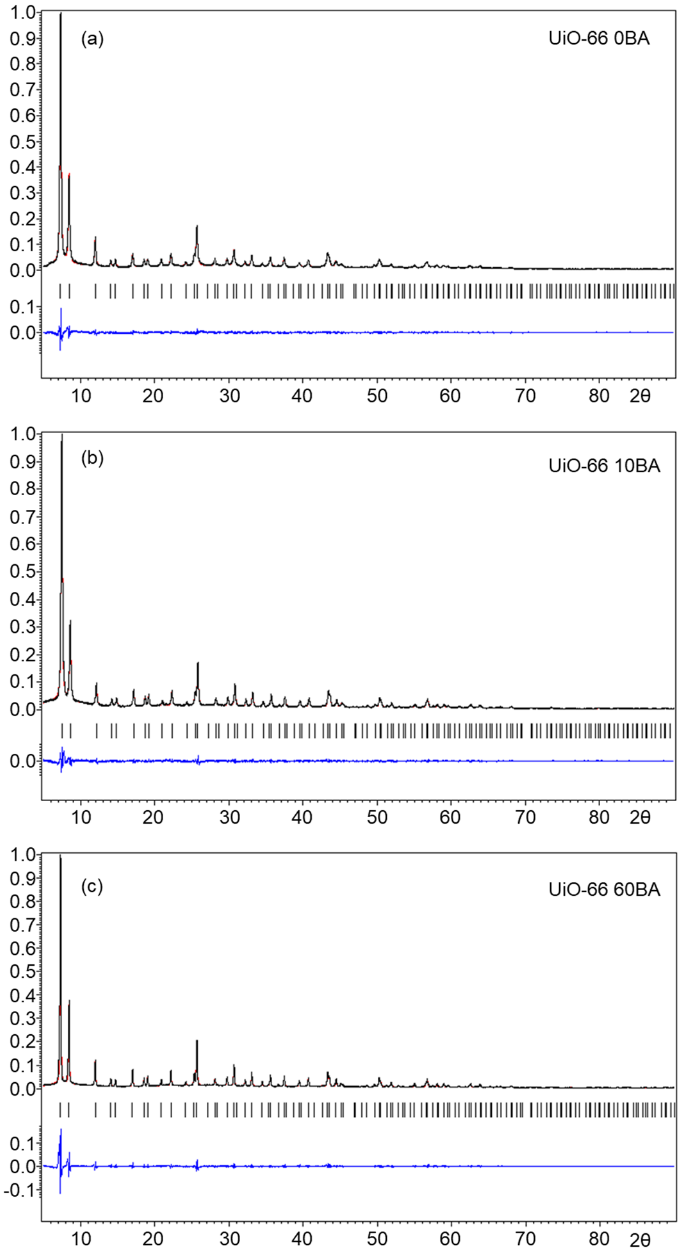

Figure S1. XRD patterns of UiO-66-0BA (a), UiO-66-10BA (b) and UiO-66-60BA (c) samples. Black profiles in each part represent experimental data; red plots are refined results, and the difference between experimental and calculated data is shown at the bottom of each part as a blue line. Vertical black lines point positions of Bragg peaks.

N_2 adsorption

We applied the BET (Brunauer-Emmett-Teller) equation to N_2 adsorption isotherms to calculate the specific surface areas. The experimental N2 adsorption isotherms are presented in the main text (Figure 2b). These data were plotted on a graph with relative pressure (P/P0) on the x-axis and the quantity of adsorbed N2 (cm3/g) on the y-axis. We identified the linear range of the N2 adsorption isotherm. This range typically corresponds to intermediate relative pressure. However, when calculating the BET specific surface area of MOFs, the optimal relative pressure range for selecting data points typically differs from the classical range used for other porous materials. This difference arises due to the unique characteristics of MOFs. In the case of MOFs, it is generally recommended to choose a relative pressure range using a list of criteria [1-3]. MOFs typically exhibit high specific surface areas, allowing for forming of a well-defined monolayer of gas molecules on their surfaces. By focusing on the low-pressure range, where monolayer adsorption dominates, the BET equation can be applied more accurately, as it assumes the formation of a single layer of adsorbate molecules. The choice of the optimal relative pressure range may vary depending on the specific MOF material and its characteristics.

Using the linear range data points, we applied linear regression to determine the slope (S) and y-intercept (Y_{int}) of the BET plot. We plotted the data on the 1/[Q(Po/P - 1)] y-axis against P/P0 on the x-axis. Q represents the quantity of adsorbed N_2 (cm^3/g STP). The slope (S) is related to the monolayer adsorption capacity, and Y_{int} is associated with adsorption energy.

The specific surface area (SSA) was calculated using the following equation:

$$SSA = \frac{CSA \cdot 6.023 \cdot 10^{23}}{22414 \cdot 10^{18} \cdot (S + Y_{int})},$$

$$C = \frac{S + Y_{int}}{Y_{int}},$$

$$Qm = \frac{1}{S + Y_{int}}.$$

SSA - specific surface area;

CSA – N_2 molecular cross-sectional area (0.1620 nm^2),

S – slope;

Y_{int} - Y-intercept;

C – BET C value;

Qm - a volume of the monolayer

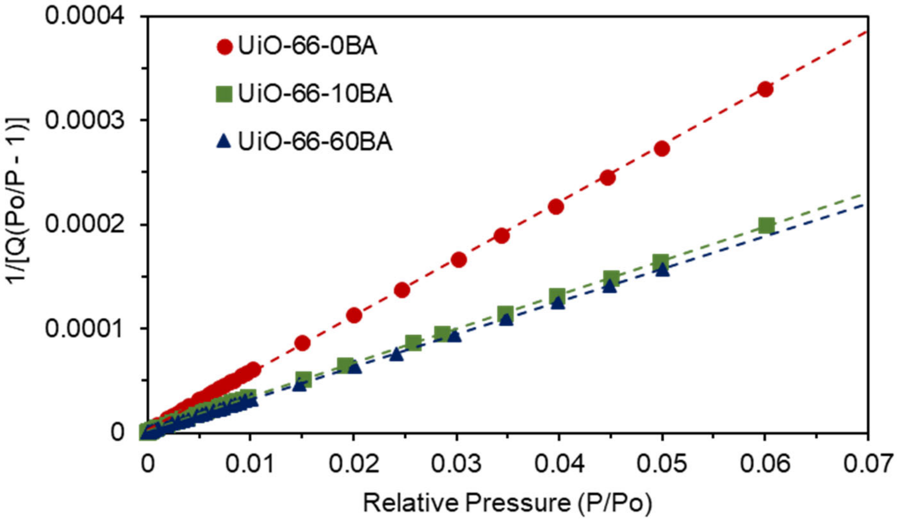

Figure S2. BET surface area plots of UiO-66-0BA (red circle markers), UiO-66-10BA (square green markers), and UiO-66-60BA (triangle navy markers).

Table S2. Some details of porosity calculations. SSA stands for specific surface area. C is constant from the BET equation.

Sample	BET calculations			Pore volume, cm^3/g	t-plot	
	SSA, m^2/g	C	Correlation Coefficient		micropore area, m^2/g	external surface area, m^2/g
UiO-66 0BA	795	2193	0.9998729	0.33	725	70
UiO-66 10BA	1331	2450	0.9999276	0.65	1132	200
UiO-66 60BA	1392	4373	0.9999708	0.55	1273	119

Microporous materials often exhibit a characteristic Type I isotherm, which is commonly associated with monolayer adsorption on a homogeneous surface. The isotherm starts with a steep increase in adsorption at low relative pressures, indicating rapid uptake as the adsorbate molecules fill the micropores. It then reaches a plateau, indicating the formation of a monolayer on the internal surfaces of the micropores. The plateau is followed by a negligible increase in adsorption at higher relative pressures as the micropores are filled, and the adsorption is limited to the surface monolayer. The synthesized UiO-66 samples exhibited Type-I isotherms, indicating the microporous nature of their crystals. The small hysteresis loop on the isotherm of the UiO-66-10BA sample can be ascribed to the phenomenon of capillary condensation occurring between aggregated particles, where N2 gas fills the spaces in a manner similar to the way it condenses in mesopores. This phenomenon is commonly observed in powders containing uniformly sized nanoparticles, as they tend to assemble into agglomerates with cavities that simulate mesopores. Consequently, the N_2 adsorption-desorption isotherm exhibits a hysteresis loop, typically associated with the capillary condensation of N_2 gas within mesopores. The UiO-66-0BA sample consists of nanoparticles that have agglomerated, with one crystal forming on the edge of another. As a result, there is minimal space between the particles, leading to dominant adsorption in micropores. This dominance in micropore adsorption determines the shape of the isotherm. The UiO-66-60BA sample is composed of polydisperse, larger microcrystals. In this case, the only available spaces for N2 adsorption are the micropores found within these microcrystals.

TGA

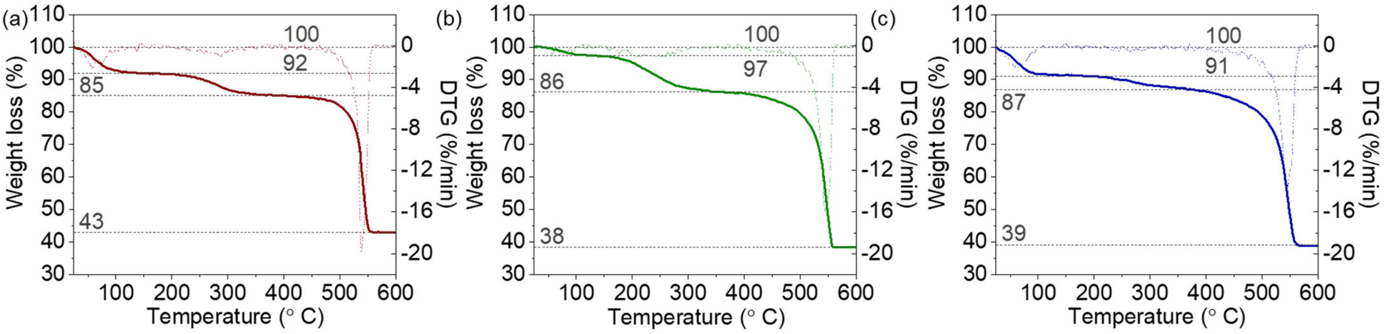

Figure S3. TGA and DTG curves of UiO-66-0BA (a), UiO-66-10BA (b), and UiO-66-60BA (c) samples.

Table S3. TGA data

Sample	Data according to $Zr_6O_4(OH)_4(BDC)_6$ formula unit		Data according to TGA	
	Weight loss, %	Molar mass	Weight loss, %	Molar mass
UiO-66-0BA	55.6	1664	49.4	1461.4
UiO-66-10BA			55.8	1673.2
UiO-66-60BA			55.2	1649.3

$$WL = \frac{M(\mathrm{Zr_6O_4(OH)_4(BDC)_6}) - 6 \cdot \mathrm{M(ZrO_2)}}{M(\mathrm{Zr_6O_4(OH)_4(BDC)_6})} \cdot 100\%$$

$$WL = \frac{1664 - 6 \cdot 123.2}{1664} \cdot 100\% = 55.6$$

$$WL\ (\mathrm{UiO-66-0BA}) = \frac{85 - 43}{85} \cdot 100\% = 49.4$$

$$WL\ (\mathrm{UiO-66-10BA}) = \frac{86 - 38}{86} \cdot 100\% = 55.8$$

$$WL\ (\mathrm{UiO-66-60BA}) = \frac{87 - 39}{87} \cdot 100\% = 55.2$$

$$M(\mathrm{UiO-66-0BA}) = \frac{100 \cdot 6(ZrO_2)}{100 - WL} = \frac{100 \cdot 6 \cdot 123.2}{100 - 49.4} = 1461.4$$

$$M(\mathrm{UiO-66-10BA}) = \frac{100 \cdot 6(ZrO_2)}{100 - WL} = \frac{100 \cdot 6 \cdot 123.2}{100 - 55.8} = 1673.2$$

$$M(\mathrm{UiO-66-60BA}) = \frac{100 \cdot 6(ZrO_2)}{100 - WL} = \frac{100 \cdot 6 \cdot 123.2}{100 - 55.2} = 1649.3$$

IR

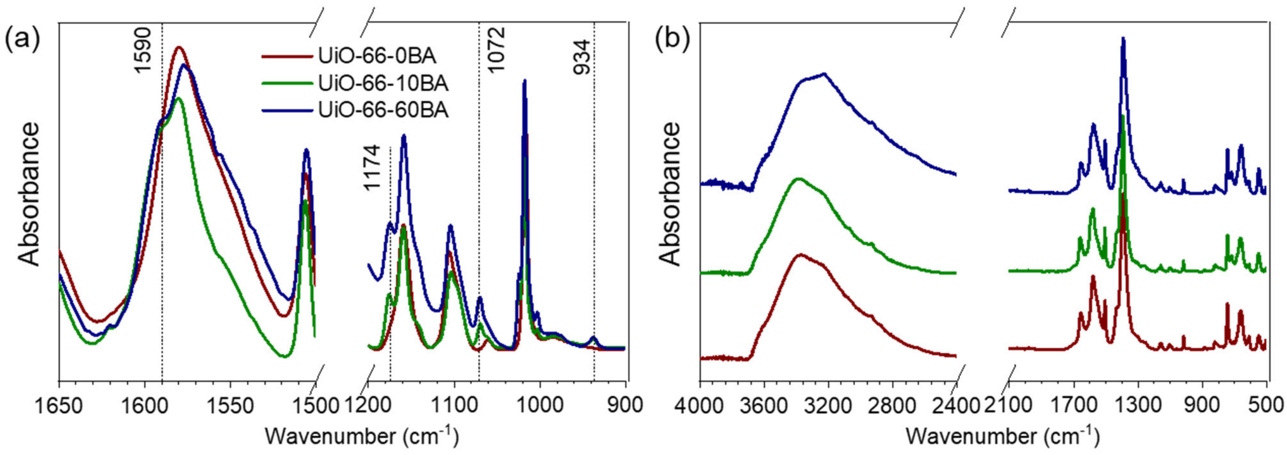

Figure S4 Selected regions of IR spectra of UiO-66-0BA, UiO-66-10BA, and UiO-66-60BA samples: 900-1200 and 1500-1650 cm-1 (a), 500-2100 and 2400-4000 (b).

NMR

The NMR spectra were recorded on a Bruker AVANCE NEO 400 MHz spectrometer with proton operating frequency 400.13 MHz equipped 5 mm iTBO triple resonance broadband probehead (BBF/H/F/D). The spectra were measured without the sample spinning at a temperature of 298 K. All spectra NMR spectra for quantitative analysis were measured with the standard 1D pulse sequence using the following experimental parameters, optimized for quantitative measurements: 30◎ hard excitation pulse, spectral width 10 ppm, 64K data points in the time domain, 16 scans and a relaxation delay of 30 s to ensure complete relaxation for all signals. The spectra were Fourier transformed after zero filling to 128 K, giving a digital resolution in the frequency domain of 0.03 Hz/pt. Careful manual phase and baseline corrections were performed prior to signal integration.

Prior to measurements, UiO-66 samples were dissolved in a deuterated alkali medium (1 M NaOD in D_2O) according to the original technique [4]. The powder was loaded into an alkaline solution, mixed properly, and held for 24 h. After this, the suspension was centrifugated and separated from the solid part. All organic components after this preparation were transferred to the liquid part as corresponding sodium salts and used for NMR measurements. White precipitate contained hydrated ZrO_2.

The 1H NMR spectrum of UiO-66 samples exhibited signals that correspond to all protons of BDC and benzoate molecules, which are indicated by Latin letters in Figure S5. Based on the integral areas of the signals of 1,4-benzene dicarboxylate (7.66 ppm, 4H) and benzoate (7.28 – 7.22 ppm, 2H, meta protons; 7.37-7.29 ppm, 1H, H-para; 7.68 – 7.62 ppm, 2H, ortho-protons) the following relative molar ratios of the two components were calculated. In the UiO-66-0BA sample, only 1,4-benzene dicarboxylate was detected. UiO-66-10BA and UiO-66-60BA samples contained both the dicarboxylate and the benzoate ions in a molar ratio of 1.95:1 and 1.68:1, respectively.

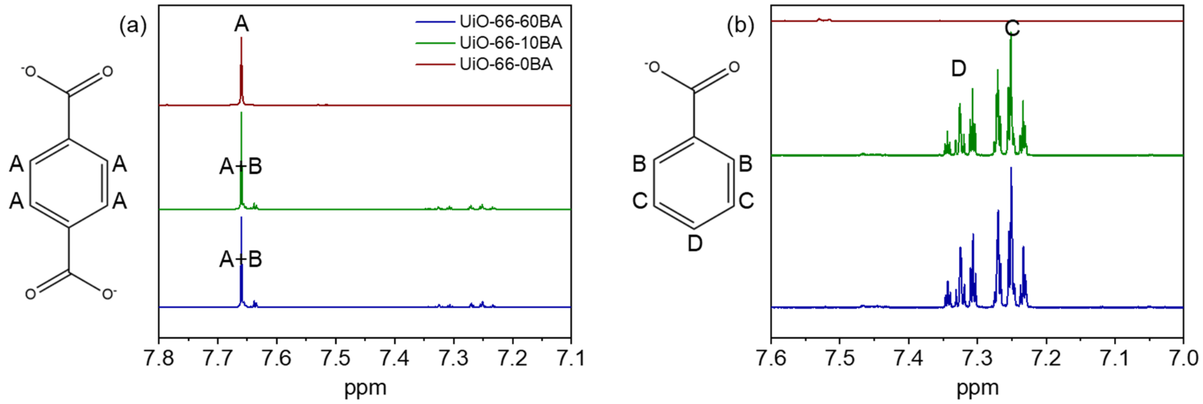

Figure S5. Fragments of the 1H NMR spectra in the region of aromatic protons of UiO-66 (a), UiO-66 10BA (b), UiO-66 60BA (c) samples. Part (b) represents a magnification of the region 7.0-7.6 ppm.

FTIR, activation

As-synthesized samples were heated in a dynamic vacuum, and FTIR spectra were measured at various temperatures from room temperature to 275 °C.

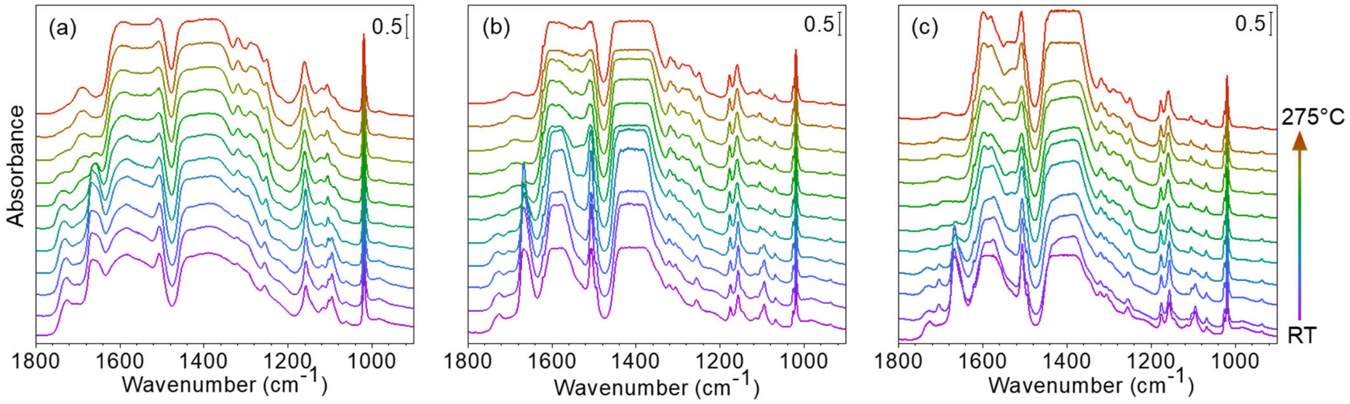

Figure S6. FTIR spectra of UiO-66 (a), UiO-66 10BA (b), UiO-66 60BA (c) samples registered after evacuation at increasing temperatures: from room temperature (bottom spectra) up to 275 °C (top spectra) in a dynamic vacuum. Spectra are provided in the 1800-900 cm^{-1} region.

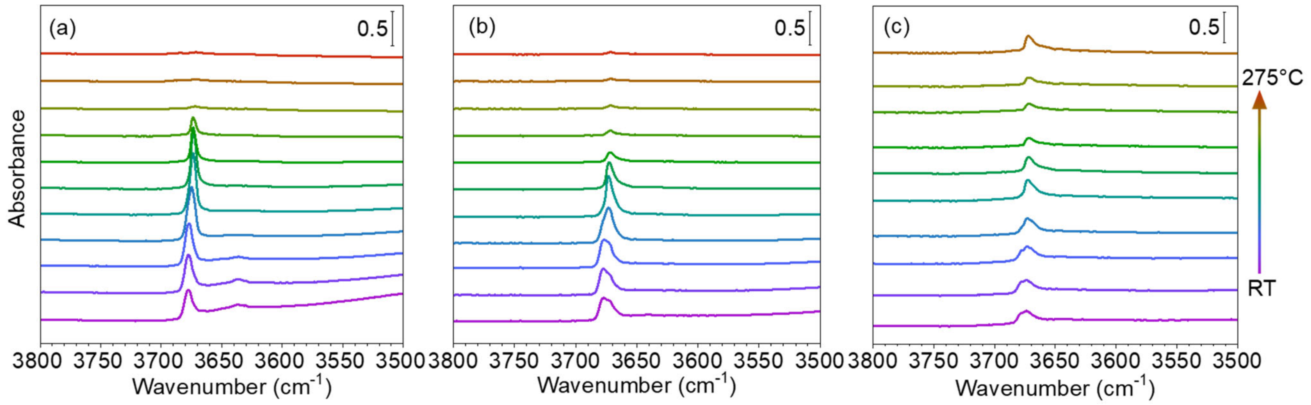

Figure S7. FTIR spectra of UiO-66 (a), UiO-66 10BA (b), UiO-66 60BA (c) samples registered after evacuation at increasing temperatures: from room temperature (bottom spectra) up to 275 °C (top spectra) in a dynamic vacuum. Spectra are provided in the 3800-3500 cm^{-1} region.

A band at 1729 cm^{-1} was observed in the spectra of all samples after the first evacuation at room temperature. Heating and outgassing led to its disappearance, indicating evacuation or transformation of the respective species. This band was assigned to hydrogen-bonded carboxylic acid [5]. We suppose that terephthalic acid residuals were trapped inside pores or covered MOF particles with one end of -COOH carboxylic groups and bonded with another end to the Zr cluster. In good agreement with this, the most pronounced peak at 1729 cm^{-1} was observed on the UiO-66-0BA spectrum, indicating a higher concentration of BDC linker. UiO-66-10BA sample contained less terephthalic acid residuals due to the competition of BDC with the modulator in the crystallization process. The UiO-66-60BA sample has fewer BDC residuals due to higher BA concentration. The activation at 250 °C gave rise to a new band at 1690 cm^{-1}, while rehydration resulted in its loss. This process is completely reversible and therefore was assigned to the process of UiO-66 dehydration (see SI). Spectra of UiO-66-10 and UiO-66-60 samples after rehydration also contained a band at 1703 cm^{-1}. This band was detected only on the spectra of samples obtained with the BA modulator. It corresponds to the C=O vibrations of BA protonated groups [6]. However, this band is also sensitive to the dehydration process and corresponded exclusively to rehydrated samples with bridge μ_3-OH groups (Figure S8).

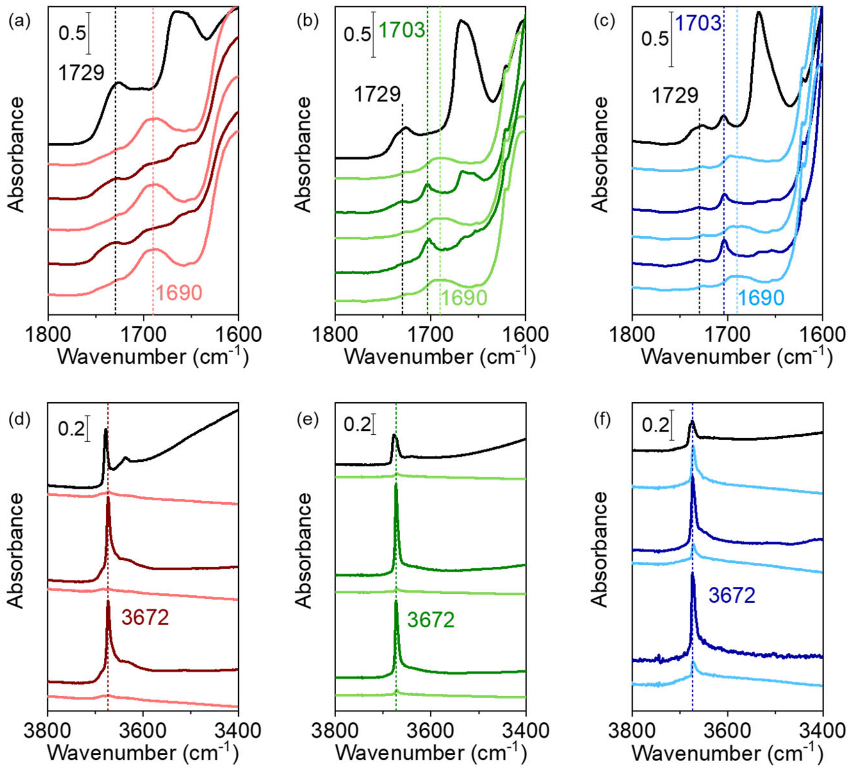

Figure S8. FTIR spectra of UiO-66-0 (a, d), UiO-66-10 (b, e), and UiO-66-60 (c, f) samples evacuated at room temperature (black lines), at 250 °C (light lines), rehydrated and evacuated at room temperature (dark lines) in a dynamic vacuum. Spectra are provided in the regions 1800-1600 cm^{-1} (a-c) and 3800-3400 cm^{-1} (d-f).

FTIR CO

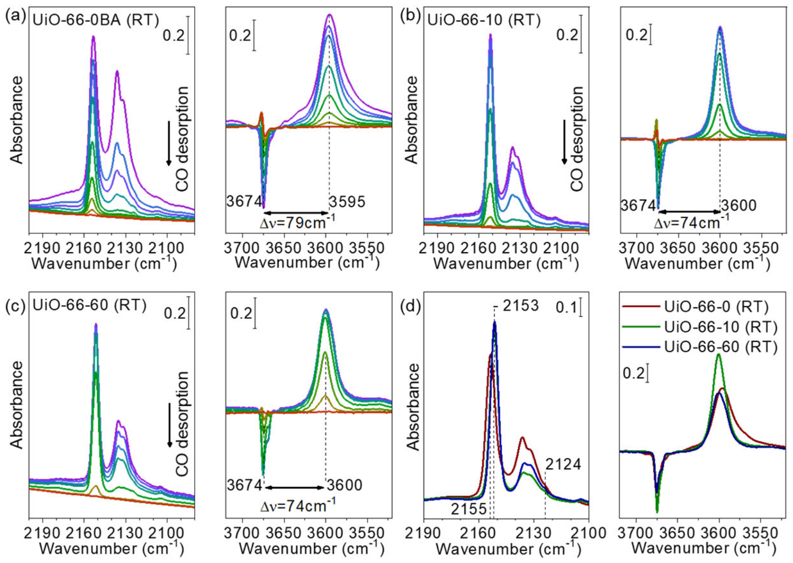

Figure S9. FTIR spectra of UiO-66-0BA (a), UiO-66-10BA (b), and UiO-66-60BA (c) samples after introduction of CO (5 mbar), followed by stepwise evacuation. Before the experiment, samples were evacuated at RT. Part (d) represents spectra of UiO-66-0BA, UiO-66-10BA, and UiO-66-60BA samples in the presence of 1 mbar of CO. Spectra are provided in the regions 2200-2080 cm^{-1} (left parts) and 3800-3500 cm^{-1} (right parts).

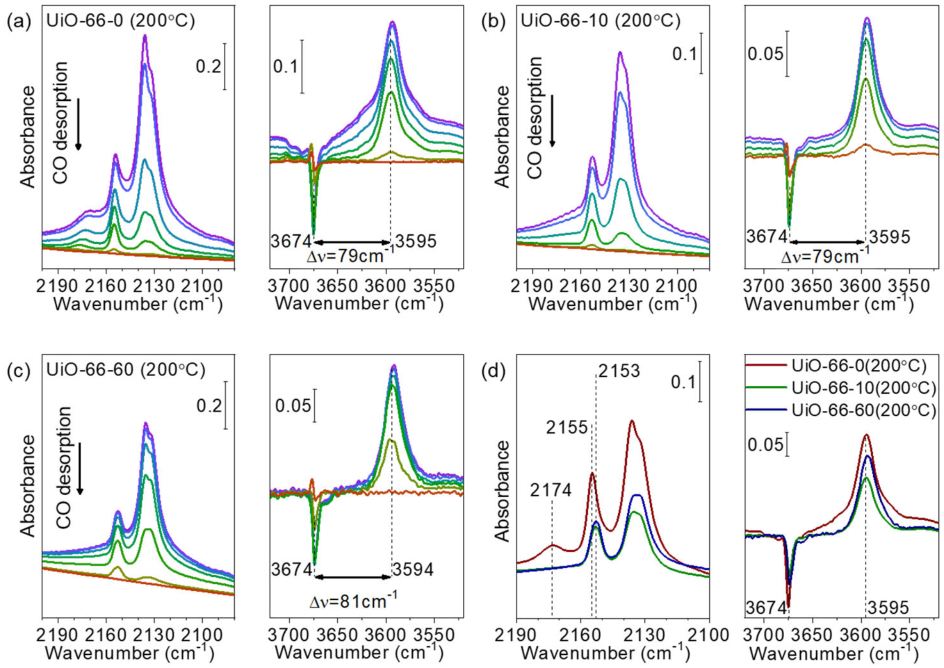

Figure S10. FTIR spectra of UiO-66-0BA (a), UiO-66-10BA (b), and UiO-66-60BA (c) samples after the introduction of CO (5 mbar), followed by stepwise evacuation. Before the experiment, samples were evacuated at 200 °C. Part (d) represents spectra of UiO-66-0BA, UiO-66-10BA, and UiO-66-60BA samples in the presence of 1 mbar of CO. Spectra are provided in the regions 2200-2080 cm^{-1} (left parts) and 3800-3500 cm^{-1} (right parts).

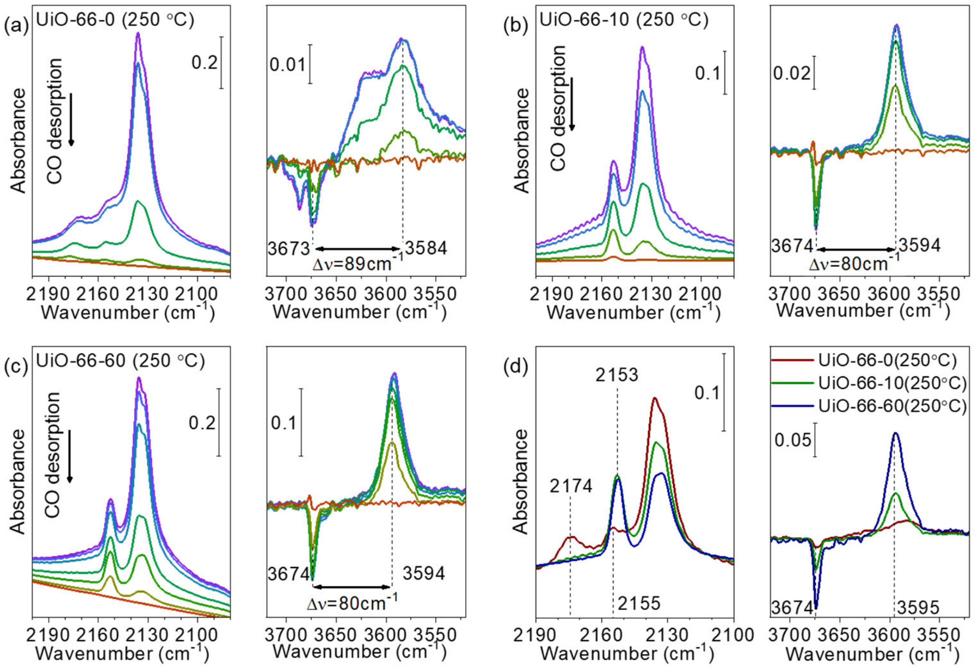

Figure S11. FTIR spectra of UiO-66-0BA (a), UiO-66-10BA (b), and UiO-66-60BA (c) samples after the introduction of CO (5 mbar), followed by stepwise evacuation. Before the experiment, samples were evacuated at 250 °C. Part (d) represents spectra of UiO-66-0BA, UiO-66-10BA, and UiO-66-60BA samples in the presence of 1 mbar of CO. Spectra are provided in the regions 2200-2080 cm^{-1} (left parts) and 3800-3500 cm^{-1} (right parts).

FTIR, CD_3CN

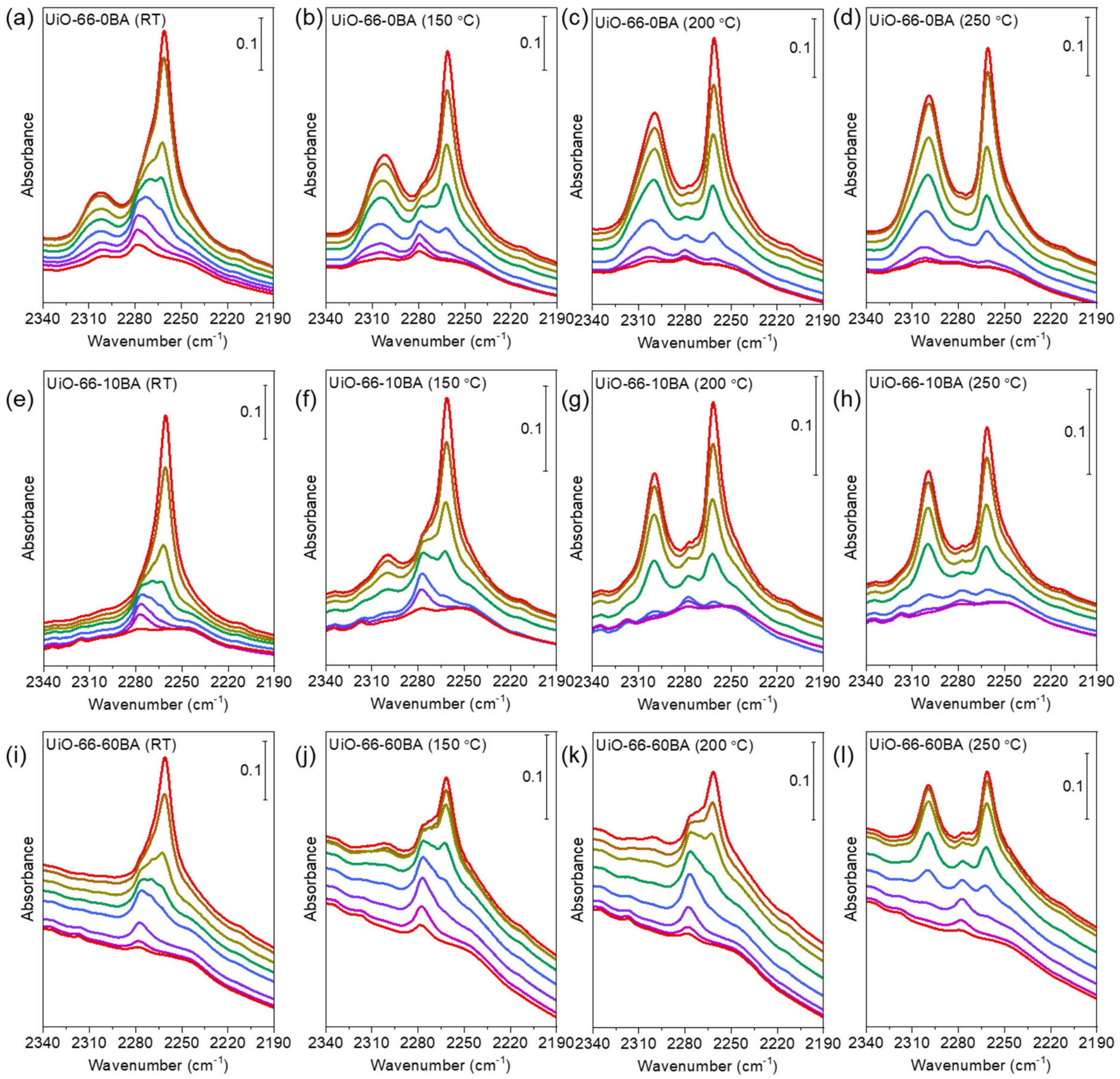

Figure S12. FTIR spectra of UiO-66-0BA (a-d), UiO-66-10BA (e-h), and UiO-66-60BA (i-l) samples after introduction of 5 mbar of CD_3CN and subsequent stepwise evacuation. Before the experiment, samples were evacuated at RT (a, e, i), 150 °C (b, f, j), 200 °C (c, g, k), and 250 °C (d, h, l).

References

1. Rouquerol, J.; Llewellyn, P.; Rouquerol, F. Is the BET equation applicable to microporous adsorbents? **2007**, *160*, 49-56, doi:10.1016/S0167-2991(07)80008-5.
2. Walton, K.S.; Snurr, R.Q. Applicability of the BET method for determining surface areas of microporous Metal–organic frameworks. **2007**, *129*, 8552-8556, doi:10.1021/ja071174k.
3. Ambroz, F.; Macdonald, T.J.; Martis, V.; Parkin, I.P. Evaluation of the BET theory for the characterization of meso and microporous MOFs. **2018**, *2*, 1800173, doi:https://doi.org/10.1002/smtd.201800173.
4. Shearer, G.C.; Chavan, S.; Bordiga, S.; Svelle, S.; Olsbye, U.; Lillerud, K.P. Defect engineering: Tuning the porosity and composition of the Metal-organic framework UiO-66 via modulated synthesis. *Chem. Mater.* **2016**, *28*, 3749-3761, doi:10.1021/acs.chemmater.6b00602.
5. Ragon, F.; Campo, B.; Yang, Q.; Martineau, C.; Wiersum, A.D.; Lago, A.; Guillerm, V.; Hemsley, C.; Eubank, J.F.; Vishnuvarthan, M.; et al. Acid-functionalized UiO-66(Zr) MOFs and their evolution after intra-framework

cross-linking: structural features and sorption properties. *J. Mater. Chem. A* **2015**, *3*, 3294-3309, doi:10.1039/C4TA03992K.

6. Atzori, C.; Shearer, G.C.; Maschio, L.; Civalleri, B.; Bonino, F.; Lamberti, C.; Svelle, S.; Lillerud, K.P.; Bordiga, S. Effect of benzoic acid as a modulator in the structure of UiO-66: An experimental and computational study. *J. Phys. Chem. C* **2017**, *121*, 9312-9324, doi:10.1021/acs.jpcc.7b00483.
